# Discovery and computational characterization of ZIKV envelope-targeted peptides from a subtractive phage display library

**DOI:** 10.1371/journal.pone.0341602

**Published:** 2026-01-29

**Authors:** Mirna Burciaga-Flores, Javier Wong-Romero, Darwin Elizondo-Quiroga, Eréndira Villalobos-Sánchez, Abel Gutiérrez-Ortega, Tanya A. Camacho-Villegas, Sergio A. Águila

**Affiliations:** 1 Centro de Nanociencias y Nanotecnología, Universidad Nacional Autónoma de México (CNyN-UNAM), Ensenada, Baja, California, México; 2 Unidad de Biotecnología Médica y Farmacéutica, Centro de Investigación y Asistencia en Tecnología y Diseño del Estado de Jalisco (CIATEJ), Guadalajara, Jalisco, México; CEA, FRANCE

## Abstract

The Zika virus (ZIKV) poses a significant public health threat, and developing highly specific diagnostic and therapeutic agents that can distinguish it from other flaviviruses remains a critical challenge. To address this, we utilized a phage display library with a strategic subtractive panning approach against the ZIKV envelope protein (ZIKV-pE). This method identified eight linear peptides with high binding ability for ZIKV-pE. Enzyme-linked immunosorbent assay (ELISA) confirmed that these peptides recognized ZIKV-pE with statistical significance compared to a bovine serum albumin (BSA) control. To elucidate the binding mechanisms, we performed molecular docking and molecular dynamics (MD) simulations. Computational analysis identified peptides R3Z15, R3Z09, R2Z05, and R3Z02 as the top candidates based on binding free energy calculations. The simulations revealed that these peptides bind specifically to the DIII domain of ZIKV-pE primarily via electrostatic interactions and form stable complexes over 300 ns of MD simulation. Our work identifies specific, high-affinity peptide binders to ZIKV-pE. It provides a structural basis for their selectivity, positioning them as promising candidates for the development of precise ZIKV diagnostics and targeted therapeutics.

## Introduction

The Zika virus (ZIKV) has emerged as a major global health threat due to its association with severe neurological complications, including microcephaly in newborns and Guillain-Barré syndrome in adults [1–2]. Crucial to the ZIKV’s life cycle is its envelope protein (ZIKV-pE), which plays a crucial role in host cell recognition and viral entry, making it a key target for therapeutic and diagnostic advancements [3–4]. However, high cross-reactivity with other flaviviruses hinders accurate detection and targeted intervention, complicating efforts to distinguish ZIKV from closely related pathogens [5]. To address these challenges, molecular targets such as the envelope protein (pE), the non-structural proteins NS1 and NS5 have been explored for their potential in diagnostics and antiviral strategies [6–7]. The ZIKV-pE consists of 90 dimers that form the virus’s icosahedral structure. It is divided into three domains (DI, DII, and DIII). The fusion loop (FL) is located in DII, while the immunogenic DIII domain elicits potent neutralizing antibodies [8–10]. However, studies in convalescent individuals have shown that antibodies targeting DI and DII exhibit lower neutralizing potency and high cross-reactivity [11–13]. Current strategies for ZIKV-pE recognition and neutralization, including polyclonal (pAbs) and monoclonal antibodies (mAbs), are hindered by limited selectivity and high cross-reactivity among flaviviruses [14–16]. The structural conservation of ZIKV-pE with other flaviviruses further complicates the development of targeted therapies and diagnostics [17]. This impediment could be overcome using phage display technology, which has emerged as a powerful tool for the rapid identification of high-affinity peptides or antibodies from synthetic or semi-synthetic libraries, enabling the selection of candidates with strong binding capabilities to specific molecular targets [18–21]. For example, monoclonal antibodies (mAbs) in various formats have thus been obtained: single variable domain on heavy chain (VHH) antibodies, fragment antigen-binding region (Fab), complete IgGs, single-chain variable fragment (scFv), and they have been successfully employed against viruses such as chikungunya, Ebola, and hepatitis [22–24]. Many of these antibody formats have been selected using the phage display technology.

Recently, phage subtraction, which utilizes viral antigens to minimize cross-reactivity, has improved the phage display technique. This method selectively isolates and amplifies phages that inherently exhibit specificity toward the target antigen (e.g., viral proteins), thereby enhancing the precision of the selected clones. For instance, in the challenging context of flaviviruses where cross-reactivity is common, subtractive panning has been successfully employed to isolate serotype-specific antibodies against Dengue virus non-structural protein 1 (NS1), demonstrating its efficacy in achieving high specificity among closely related viral targets [25]. However, a significant limitation of monoclonal antibodies (mAbs) is their inability to access epitopes that are not exposed on the protein surface. This issue is particularly relevant in ZIKV infections due to the inherent conformational dynamics of the viral envelope proteins, a process sometimes described as “breathing”, which involves transient rearrangements that expose or conceal epitope [26]. These limitations can be overcome by using linear peptides that target hidden or less accessible epitopes. Additionally, computational approaches such as molecular docking and molecular dynamics (MD) provide a powerful strategy for precisely characterizing and screening peptide candidates, allowing for detailed analysis of their binding interactions and stability with target epitopes. This study examines the crucial role of integrating experimental and computational methods to elucidate the intricate interplay between selectivity and stability, thereby informing the rational design and development of more effective peptide-based diagnostics and therapeutics [27–29].

Herein, we employed an innovative approach that integrates phage display technology with molecular dynamics (MD) simulations to identify and characterize linear peptides targeting ZIKV-pE. Three rounds of biopanning using a subtractive strategy were performed to isolate several unique 20-amino-acid peptides from a synthetic library. Their specific recognition of ZIKV-pE was demonstrated in ELISA assays. To gain deeper insight into molecular interactions, molecular docking was combined with extensive MD simulations, which enabled a detailed evaluation of the stability and binding dynamics of the peptide-ZIKV-pE complexes. This synergistic combination of experimental and computational techniques enhances the precision of peptide selection, providing a robust framework to understand the structural basis of peptide-target interactions. Our findings establish a solid foundation for the development of peptide-based antiviral agents and diagnostic platforms that selectively target ZIKV-pE. This integrated approach presents a promising and innovative strategy for addressing the challenges posed by ZIKV and related flaviviruses, highlighting the potential of combining high-throughput experimental methods with advanced computational tools in antiviral research.

## Results

### Biopanning of specific peptides for ZIKV-pE recognition

**Fig 1:**
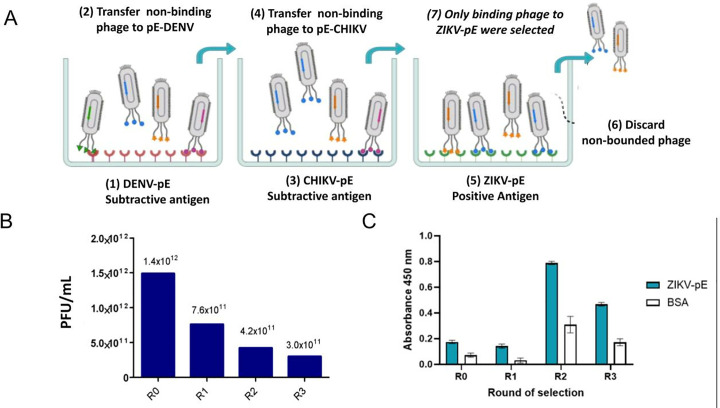
Phage-display for selection of anti-ZIKV-pE peptides. (A) Recombinant antigens from the envelope proteins of DENV, CHIKV, and ZIKV were adsorbed onto a 96-well plate. The Trico-20 phage library was exposed to each antigen in turn. The first two wells were used for subtractive purposes to deplete binders against DENV-pE and CHIKV-pE. Only binders in the last tube against the ZIKV-pE were eluted for further characterization. (B) Phage titer was expressed in plaque-forming units per milliliter (PFU/mL) for each selection round. (C) Specificity of selected peptide-displaying phages for related ZIKV-pE or BSA (used as non-related viral control) were immobilized in wells for ELISA. Bound phages were detected using an antibody against M13 phage. Values mean standard deviation (n = 3). During the second and third rounds of panning a peptide library, we observed an enrichment of bacteriophages displaying specific peptides. This enrichment, indicating increased specificity, was more pronounced in the second round.

To minimize potential cross-reactivity with phylogenetically related viruses, we employed a subtractive phage display strategy using a TriCo-20 library (Creative Biolabs). This involved pre-adsorbing the library against the envelope proteins of dengue (DENV) and chikungunya (CHIKV) viruses (Fig 1A). This subtractive approach was used to preferentially isolate peptides specific to ZIKV-pE, thereby minimizing the recovery of clones that cross-react with other flaviviruses. Three iterative rounds of selection were performed, each involving the subtraction of phages that bound to the envelope proteins of DENV and CHIKV. Phage titers were quantified after each round, revealing a progressive decline from 7.6 × 10¹¹ plaque-forming units per milliliter (PFU/mL) in the first round to 4.2 × 10¹¹ PFU/mL in the subsequent rounds (Fig 1B), reflecting the elimination of phages with low specificity or binding ability for ZIKV-pE. Despite this reduction, phages displaying anti-ZIKV-pE peptides were successfully amplified (Fig 1C). From the second and third rounds, 20 clones (5 and 15 isolated clones, respectively) were randomly selected for further characterization.

### DNA sequence analysis of randomly selected phage clones

The screening of the 20 phage clones involved DNA extraction and amplification, followed by integrity and purity assessment to confirm their suitability for subsequent sequence analyses (S1 Fig. and S1 File). Of the 20 phage clones, eight sequences of interest were identified (Table 1).

**Table 1:** In silico analysis of peptides obtained by phage-display against ZIKV-pE.

Peptide	Sequence	Length (aa)	MW (Da)	pI	Hydrophobicity index	Half-Life (hours)	Pep-Fold4.0 Energy Score (Kcal/mol)
R2Z03	FQPKWVARPAIQQHIVKNAE	20	2360.75	9.99	33.28	1.1	− 36.2883
R2Z05	MYKAPERHGQQPDWSKHQT	19	2324.56	8.29	17.23	30	−32.1178
R3Z02	EAHLFSHSNWQVMSLSQSES	20	2304.48	5.23	38.46	1	− 43.7656
R3Z05	SWTKPVSHEHSQNLGTWPMT	20	2323.57	6.66	34.83	1.9	−26.8178
R3Z07	YPGSPTQYPSSMHEYHSSSE	20	2271.36	5.23	20.99	2.8	−28.492
R3Z09	SHIQPYQMYPQAFFMGKAT	19	2245.60	8.24	38.36	1.9	−31.4214
R3Z13	SHVDFTIYRKMVDMKHSRHTE	21	2617.98	8.29	33.57	1.9	−40.0077
R3Z15	GFSHPLSKTETVFAQQSRAA	20	2162.39	8.75	29.24	30	−31.1924

**Fig 2:**
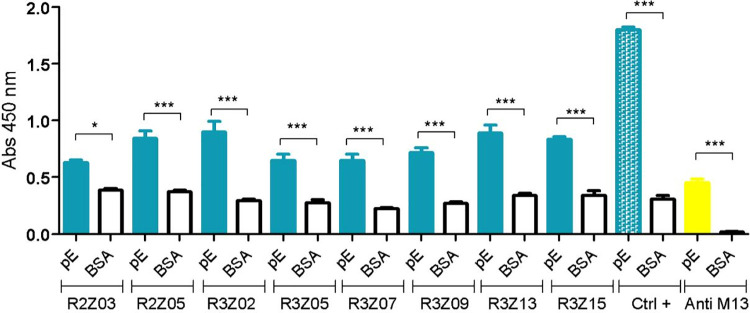
Screening via ELISA assay of phage clones displaying peptides with binding capacity to ZIK-pE. Recombinant ZIKV-pE was used as an antigen. All selected phages displaying peptides were challenged with ZIKV-pE, and the ELISA assay was performed with an Anti-M13-HRP antibody as the secondary antibody. The positive control (Ctrl +) consisted of a commercial monoclonal antibody against ZIKV-pE. An anti-M13 antibody was also used as a control to determine background recognition. Error bars represent standard deviations of the mean from three biological replicates. Statistical analysis was conducted using one-way ANOVA, followed by Tukey tests. Significance levels are indicated: ***p < 0.001, **p < 0.01 y *p < 0.05. Brackets indicate statistically significant p-value comparisons for the bars depicting the means. The experiment was performed with n = 3 biological replicates.

The physical and chemical parameters of these peptides were computed using the ProtParam tool from the ExPASy server (https://web.expasy.org/protparam/) [30]. The eight peptide sequences differed in sequence, length (19 to 21 aa), molecular weight (21.6 to 26.1 kDa), and theoretical isoelectric point (pI) (5.23 to 9.99). The enzyme-linked immunosorbent assay (ELISA) was performed using recombinant ZIKV-pE protein as the antigen, and bovine serum albumin (BSA) as a negative control to assess the ZIKV-pE-specific binding of the selected peptides. The experiment was conducted with three biological replicates to ensure reliability and reproducibility. Our results showed that the eight selected phage-displaying peptide clones recognized the ZIKV-pE with a statistically significant difference (***p < 0.001) compared with the 3% BSA negative control (Fig 2 and S1 File), indicating specificity. The selected peptides were R2Z03, R2Z05 from round 2, and R3Z02, R3Z05, R3Z07, R3Z09, R3Z13, R3Z15 from round 3.

### Computational assessment of peptide stability and theoretical half-life

For potential diagnostic or therapeutic use, the selected peptides must exhibit favorable stability profiles, including structural integrity under storage conditions and resistance to proteolytic degradation [31]. To evaluate the selected peptides, we conducted in silico analyses of their structural stability and half-life in a human reticulocyte model as a first approach. Our stability assessment highlighted R2Z05 and R3Z15 for their optimal combination of long theoretical half-life (30 h) and moderate structural stability. In contrast, R3Z13 had the highest structural stability score and a favorable pI, but was offset by a shorter half-life (1.9 h). Further analysis is needed, such as *in vivo* pharmacokinetics assays, to confirm those results obtained in the in silico approach.

### *In silico* assays by molecular docking and molecular dynamics (MD)

The electrostatic surface mapping of the ZIKV-pE, performed using the Adaptive Poisson-Boltzmann Solver (APBS), provided essential insights into the protein’s electrostatic potential, which is pivotal to understanding its interaction landscape, structural stability, and dynamic behavior. This analysis delineated the spatial distribution of charged regions, highlighting key areas of negative and positive electrostatic potential that mediate molecular interactions and influence conformational dynamics. Such detailed electrostatic profiling is fundamental to elucidating the mechanistic underpinnings of biological function and guiding structure-based drug design efforts. These findings are crucial for accurate molecular docking and MD simulations, as they inform predictions of binding sites, interaction mechanisms, and conformational behavior [32].

**Fig 3:**
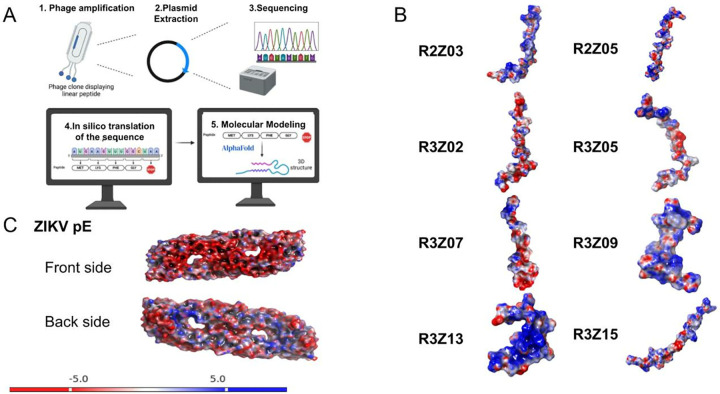
Electrostatic surface mapping of selected peptides and ZIKV-pE. (A). Experimental workflow to obtain the nucleic acid sequence that encodes the target peptides, followed by in silico translation and computational modeling. (B) Electrostatic potential surfaces of the eight selected peptides. Each peptide is labeled with its corresponding identifier (e.g., R2Z03, R2Z05). The charge distribution is depicted using the same color scheme as in (A), with blue representing positive charges, red representing negative charges, and white representing neutral regions. The peptides exhibit varying charge distributions, which may influence their predicted binding affinity to ZIKV-pE. (C) Electrostatic potential surface of the ZIKV-pE (PDB ID: 5JHM). The structure represents the soluble ectodomain (residues 1–409) of ZIKV-pE, the crystallizable form used in this study. The surface is color-coded to represent charge distribution, with blue indicating positive charge, red indicating negative charge, and white indicating neutral regions. The inner side of DII shows the highest negative charge density, while the FL and adjacent DI regions exhibit strong positive charge.

The APBS analysis of the peptide charge distribution revealed diverse electrostatic profiles, with net charges ranging from −1 to +3 (Fig 3B, Table 1). The APBS analysis of ZIKV-pE indicated a net negative charge of -7e per chain, with the inner side of domain II (DII) exhibiting the highest negative charge density (Fig 3C). In contrast, the outer side of domain III (DIII) displayed moderate negative charge density, while its inner side showed positive charge density. The fusion loop (FL) and its adjacent domain I (DI) region were characterized by the highest positive charge density (Fig 3C). Notably, the dimer exhibited an asymmetric charge distribution, with one side predominantly negative and the other tending toward a neutral-positive profile.

**Fig 4:**
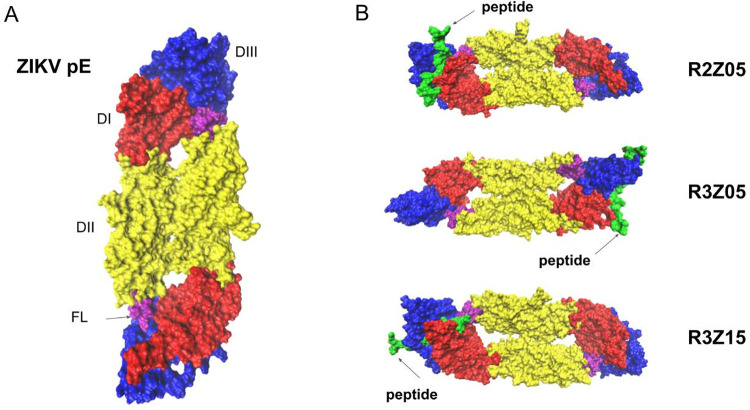
Most representative molecular dynamics (MD) frame for the peptide-ZIKV-pE complex. (A) Structure of the Zika virus envelope protein (ZIKV-pE, PDB ID: 5JHM), representing the soluble ectodomain (residues 1–409). The domains are color-coded as follows: Domain I (DI, red), Domain II (DII, yellow), Domain III (DIII, blue), and the fusion loop (FL, purple). The peptides are shown in green to highlight their binding sites on ZIKV-pE. (B) Detailed interaction of selected peptides with ZIKV-pE: R3Z15: Interacts with DI and DIII, R3Z09: Contacts DI and DII, and R2Z05: Primarily binds to DIII and DI.

**Fig 5:**
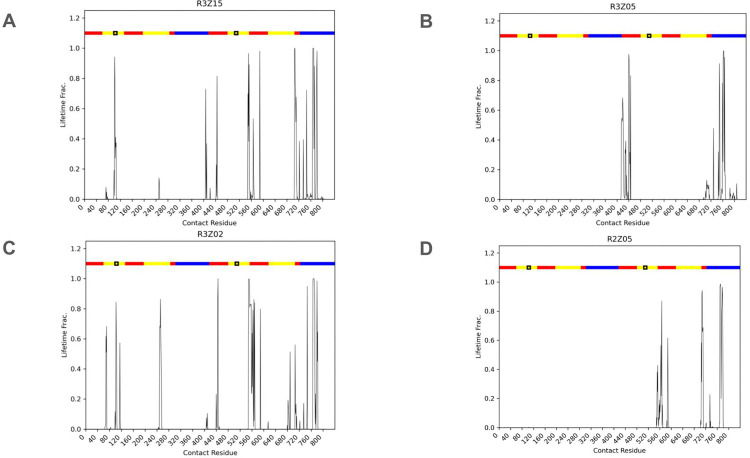
Contact lifetime of ZIKV-pE residues with linear peptide(s) across the trajectory. Contact lifetime of ZIKV-pE residues with (A) R3Z15, (B) R3Z05, (C) R3Z02, and (D) R2Z05, respectively. Residue contacts are considered within 5 Å of both molecules. The X-axis: Residues of ZIKV-pE (soluble ectodomain, residues 1–409, PDB ID: 5JHM). The domains are color-coded as follows: Domain I (DI, red), Domain II (DII, yellow), Domain III (DIII, blue), and the fusion loop (FL, purple), and the Y-axis: Fraction of the 300 ns MD trajectory during which a residue is in contact with the peptide. A value of 1.0 indicates continuous contact throughout the simulation, while lower values indicate transient or intermittent interactions.

Molecular docking and MD simulations were employed to elucidate the interaction mechanisms between the peptides and ZIKV-pE, corroborating *in vitro* findings. Docking studies demonstrated high accuracy, with minimal peptide displacement observed after 300 ns molecular dynamics runs, yielding final poses consistent with the initial docking results (Fig 4). Our computational analysis suggests most peptides exhibited a binding preference for DIII (R2Z03, R2Z05, R3Z05, R3Z07, R3Z13, R3Z15). The contact map (Fig 5, and S5 Fig.) and binding free energy calculations (Table 2) using Molecular Mechanics/Poisson-Boltzmann Surface Area (MM/GPSA) [33] further supported these observations. MM/GBSA results aligned with *in vitro* data, confirming successful peptide binding to ZIKV-pE. Peptides R3Z15, R3Z09, R2Z05, R3Z07, and R3Z02 emerged as the most potent binders, with binding free energies of −69.2, −62.1, −60.27, −56.56, and −55.62 kcal/mol, respectively.

**Table 2:** MM/GBSA binding free energy composition. Major contributions are highlighted in bold text. MM contributions: Molecular mechanics contributions represent the internal and intermolecular energies of the molecular system in a vacuum or in an explicit solvent. PB contributions: The Poisson-Boltzmann equation is used to represent solvation effects, particularly electrostatic solvation, utilizing an implicit solvent model. It is calculated as: ΔG = ΔGMM +ΔGPB. The Cluster Fracc. The column represents the percentage of time that the cluster is present in the entire 300 ns trajectory.

System	Charge (e^-^)	ΔG_MM_ (kcal/mol)	ΔG_PB_ (kcal/mol)	ΔG (kcal/mol)	Cluster Fracc.
**R3Z15**	2	−457.6	388.4	**−69.20 ± 0.95**	0.308
**R3Z09**	2	−547.74	485.63	**−62.10 ± 0.74**	0.450
**R2Z05**	2	−433.3	373.17	**−60.27 ± 1.89**	0.355
**R3Z07**	−1	−214.13	157.59	**−56.54 ± 2.00**	0.396
**R3Z02**	−1	−10.81	−44.8	**−55.62 ± 2.48**	0.428
R3Z13	2	−281.22	248.1	−33.12 ± 0.43	0.575
R3Z05	1	−65.68	42.73	–22.95 ± 1.06	0.777
R2Z03	3	−220.96	231.96	10.99 ± 1.36	0.526

## Discussion

In this study, we successfully identified eight peptides that bind specifically to the Zika virus envelope protein (ZIKV-pE) using a subtractive phage-display strategy. Our integrated approach, combining experimental biopanning with computational simulations, not only isolated these peptides but also provided a molecular rationale for their predicted binding, highlighting their potential as tools for ZIKV detection and neutralization.

The strategic subtraction of phages binding to DENV-pE and CHIKV-pE was critical for enhancing selectivity. In this subtractive biopanning strategy, the observed decrease in the total titer of phages eluted from ZIKV-pE over successive rounds (Fig 1B) is precisely as expected. This reduction is a direct consequence of the progressive elimination of non-specific and cross-reactive phages that bound to DENV-pE and CHIKV-pE (which were subsequently discarded and not titrated against ZIKV-pE) during the initial subtraction steps, combined with the stringent washes applied in each round. This strategy effectively reduces cross-reactivity with DENV and CHIKV by narrowing the phage pool to highly specific binders for ZIKV-pE, whose enrichment is qualitatively confirmed by the increased ELISA signal in Fig 1C, rather than an increase in total phage output. This yielded a diverse set of peptides, an outcome consistent with other phage display studies, such as the selection against SARS-CoV-2 [34].

ELISA binding assays robustly confirmed specific binding these peptides, with seven out of eight clones showing statistically significant recognition of ZIKV-pE over a BSA control at a 99.9% confidence level (Fig 2). Subsequent molecular dynamics simulations revealed that the peptide-ZIKV-pE complexes reached a stable conformational state within the 300 ns simulation period, as evidenced by RMSD and RMSF analyses ( S2, S3 Figs). This stability lends credibility to the calculated binding free energies, which identified peptides R3Z15, R3Z09, R2Z05, R3Z07, and R3Z02 as the top candidates with the most favorable interaction scores, using the MM/PBSA and MM/GBSA methods.

A key finding of our computational analysis is the predicted predominant binding of these peptides to domain III (DIII) of ZIKV-pE (Figs 4, 5). DIII is a critical target for neutralizing antibodies as it mediates viral attachment and entry [35–37]. The consistent computational targeting of DIII by peptides with diverse primary sequences underscores the effectiveness of our subtractive panning strategy. This is particularly significant because DIII is the most variable domain among flaviviruses, which likely contributed to the selection of peptides with reduced cross-reactivity potential, directly addressing a key challenge in ZIKV-specific diagnostic and therapeutic development. Notably, peptides like R3Z02 and R3Z07 are predicted to position themselves near the fusion loop (FL), a region implicated in the early stages of flavivirus infection [38–40]. This suggests a potential mechanism for viral neutralization by obstructing fusion, a hypothesis that warrants future experimental validation.

While the DIII surface exhibits a net negative charge, the predicted binding of peptides with varying net charges (Table 2) indicates that interactions are driven by specific electrostatic complementarity at the binding interface rather than global charge. This may be explained by interactions with localized positive patches or conformational dynamics within DIII, highlighting the complexity of flavivirus attachment, which often involves electrostatic interactions with host glycosaminoglycans [41]. Furthermore, the small size and flexibility of our linear peptides may provide a distinct advantage over bulkier monoclonal antibodies (mAbs). They could potentially access cryptic epitopes exposed during the dynamic conformational changes of the viral envelope proteins, which involve transient rearrangements that reveal hidden surfaces, particularly on DIII [42,43]. This positions our peptides as promising candidates for targeting these vulnerable, often inaccessible sites [14].

When evaluating the therapeutic potential of the lead peptides, stability is a crucial factor. Peptides R3Z15 and R2Z05 emerge as the most promising candidates, predicted binding strength (as indicated by ELISA signal and predicted by computational methods) with an optimal theoretical half-life, making them suitable for both diagnostic and therapeutic applications. In contrast, peptides like R3Z09, R3Z07, and R3Z02, despite their strong binding ability, have shorter theoretical half-lives that may limit their therapeutic use. However, their high predicted affinity still makes them excellent candidates for rapid diagnostic assays, and their stability could be improved through future chemical modifications [44].

Although the 300 ns MD simulations indicated stable binding, the convergence of free energy estimates could be further validated in future work with extended sampling and replica simulations. The most significant limitation of this study is the lack of direct experimental validation of peptide specificity against related flaviviruses (DENV-pE and CHIKV-pE) and *in vitro* neutralization assays to validate the peptides’ functional inhibitory potential. Future work must focus on these experiments to confirm their ability to block ZIKV infection. Additionally, the peptides’ specificity over a broader panel of flaviviruses should be empirically tested. Finally, insights into the predicted binding mechanisms, such as their interactions with the fusion loop, provide a clear rationale for redesigning and optimizing these peptides to achieve even greater affinity and specificity.

## Methodology

### Subtractive phage-display method to select ZIKV-pE binding phages

This study aimed to isolate specific peptides that bind exclusively to ZIKV-pE (Fitzgerald, 30–1935) using the subtractive phage-display technique, employing a library containing independent random linear peptides (Trico-20) (Creative Biolabs Library ID TriCo-20, USA). The original protocol was modified, adding a subtraction step, as depicted in Fig 1A, to minimize cross-reactivity with DENV and CHIKV. In this step, peptide-displaying phages that bound to DENV-pE (MyBioSource, MBS596102) and CHIKV-pE (Fitzgerald, 30–1940 were selectively removed, followed by six astringent washes before eluting only those phages showing interaction strength for ZIKV-pE. The ZIKV-pE-binding phages obtained after three rounds of subtractive panning were eluted using the protocol provided by the commercial library and then used for further characterization. An Enzyme-linked immunosorbent assay (ELISA) was conducted to assess the specificity of each round of biopanning. ZIKV-pE was immobilized at a concentration of 2.5 ng/µL on a 96-well microplate overnight at 4 °C. The plate was blocked with 200 µL of 3% BSA in 1X PBS for 2 h at 37 °C. Next, 50 µL of the freshly amplified phages was added to each well, and the wells were challenged with ZIKV-pE or 3% BSA as the negative control. After each step, the wells were washed three times with 150 µL of 1X PBS containing 0.05% Tween 20. Subsequently, a horseradish peroxidase (HRP)-conjugated anti-M13 antibody (Santa Cruz Biotechnology, Sc-53004-HRP) was added at a concentration of 1 µg/mL and incubated overnight at 4 °C. Three additional washing steps followed this. Thereafter, 50 µL of 1-Step Ultra TMB solution (Thermo Scientific, 34028) was added per well. The reaction was stopped with 50 µL of 0.5 M H_2_SO_4_, and the absorbance was measured at 450 nm using an xMark Microplate Spectrophotometer (BioRad). The experiment was conducted with three biological replicates to ensure reliability.

### Screening of phage clones

A total of 20 individual phage clones were randomly chosen, 15 from round 3, and five clones from round 2. They were amplified by infecting 10 mL of *E. coli* ER2738 culture (OD600 nm ~ 0.5) and incubating overnight at 37 °C and 250 rpm. The culture was then centrifuged at 10,000 rpm and 4 °C for 15 minutes, and the resulting cellular pellet was used for DNA extraction, following the protocols provided by the Zymo Miniprep kit (Zymo Research, USA). The extracted M13KE vectors were then evaluated by electrophoresis in a 1% agarose gel stained with Sybr Safe to determine their integrity. The sequencing service offered by the Laboratorio Nacional de Biotecnología Agrícola, Médica y Ambiental (LANBAMA-IPYCIT) was commissioned to determine the nucleotide sequence of the isolated clones. The peptide sequences were analyzed *in silico* using the ProtParam tool available on the ExPASy server (https://web.expasy.org/protparam/). This tool determined key physicochemical properties, including amino acid composition, molecular weight, isoelectric point (pI), and *in vivo* half-life in mammalian reticulocytes. To evaluate the structural stability of the peptides, we used PEP-Fold 4.0 [45], a computational tool for peptide structure prediction and energy scoring, accessible at https://bioserv.rpbs.univ-paris-diderot.fr/services/PEP-FOLD4/. This tool provided energy scores for each peptide, which were used to assess their conformational stability.

#### Amplification of phage clones and enzyme-linked immunosorbent assay (ELISA).

Among the 20 selected phage clones, 8 contained unique peptide sequences (R2Z03, R2Z05, R3Z02, R3Z05, R3Z07, R3Z09, R3Z13, and R3Z15), which were subsequently re-amplified for use in binding assays. Amplification was conducted by infecting 20 mL of *E. coli* ER2738 culture (DO_600nm_ ~ 0.5). The infected culture was then incubated for 4.5 h at 37 °C and 250 rpm. Subsequently, the culture was centrifuged at 10,000 rpm and 4°C for 15 minutes. Then, 1/8 volume of 20% polyethylene glycol (PEG 8000) and 2.5 M NaCl was added to the cell supernatant, followed by overnight incubation at 4°C to precipitate the amplified phages. The phage pellet obtained was centrifuged at 10,000 rpm and 4 °C for 15 min, then resuspended in 10 mL of sterile 1X phosphate-buffered saline (PBS) (Sigma, P4417-50TAB). The resuspended phage pellet was further diluted to 1 mL with 1X PBS and sterilized by filtration through 0.20 µm PTFE filters (Merck, SLLG025SS) in a biosafety cabinet (Bio II Advance Plus, Telstar 12469–2000). Enzyme-linked immunosorbent assays (ELISA) were conducted to assess the specificity of the selected phages. ZIKV-pE was immobilized at a concentration of 2.5 ng/µL on a 96-well microplate overnight at 4 °C. The plate was blocked with 200 µL of 3% BSA in 1X PBS for 2 h at 37 °C. Next, 50 µL of the freshly amplified phages was added to each well, and the wells were challenged with ZIKV-pE or 3% BSA as the negative control. A commercial anti-ZIKV-pE monoclonal antibody (mAb) (Fitzgerald, 10–2714) was used at a concentration of 1 µg/mL and incubated overnight at 4 °C as the positive control. After each step, the wells were washed three times with 150 µL of 1X PBS containing 0.05% Tween 20. Subsequently, a horseradish peroxidase (HRP)-conjugated anti-M13 antibody (Santa Cruz Biotechnology, Sc-53004-HRP) was added at a concentration of 1 µg/mL and incubated overnight at 4 °C. Three additional washing steps followed this. Thereafter, 50 µL of 1-Step Ultra TMB solution (Thermo Scientific, 34028) was added per well. The reaction was stopped with 50 µL of 0.5 M H_2_SO_4_, and the absorbance was measured at 450 nm using an xMark Microplate Spectrophotometer (BioRad). The experiment was conducted with three biological replicates to ensure reliability.

### Characterization of the interactions by molecular docking and molecular dynamics

The computational characterization protocol consisted of three main steps: molecular docking to generate initial complexes, molecular dynamics (MD) simulations to assess stability, and finally, binding free energy calculations using frames extracted from the production MD trajectories. The crystallographic structure of ZIKV-pE was used (PDB ID: 5JHM), which represents a truncated version of ZIKV-pE (residues 1–409) lacking the stem region and the C-terminal transmembrane anchor, with a resolution of 2.0 Å. This construct corresponds to the soluble ectodomain of the E protein, which is responsible for receptor-binding and membrane fusion. The absence of the transmembrane anchor in this construct allows for more precise structural analysis and computational modeling, as it focuses on the functional ectodomain [46]. Each isolated peptide sequence (R2Z03, R2Z05, R3Z02, R3Z05, R3Z07, R3Z09, R3Z13, R3Z15) was used to model its structure. Before MD preparation, an EPS map was generated using the APBS module in Maestro, Schrödinger [47]. Afterwards, molecular dynamics simulations were performed on ZIKV-pE before generating protein-peptide complexes. Predictions from Hpepdock were used to identify the ideal starting position and orientation of the peptides relative to ZIKV-pE [48]. The server determines the optimal docking score by assessing binding poses using a scoring function that computes the binding free energy, accounting for van der Waals forces, electrostatic interactions, and desolvation effects [49,50]. The pose with the most favorable energy score, or the lowest energy score, represents the highest predicted binding affinity and was consequently selected as the best model. This model was then prepared for molecular dynamics simulations and analyzed using AMBER24 with the ff19SB force field. To enhance conformational sampling and overcome energy barriers, thereby validating the exploration of a broader range of conformational states, the systems were solvated in a water box with a padding of 12 Å using a TIP3P water model with 0.15 M Na^+^ and Cl^-^ ions.

Then, 20,000 minimization steps were performed using a conjugate gradient method, followed by an NVT heating phase using simulated annealing, which increased from 0 K to 400 K over 500 ps. Then, the temperature was held for 500 ps. After that, a cooling phase from 400 K to 310 K occurred over 500 ps. Then, NVT equilibration was run for 1 ns. Afterwards, the systems were run with Langevin dynamics in an NPT ensemble until equilibration and then completed with a 300 ns production phase. Once the MD runs were completed, the root mean square deviation (RMSD) and root mean square fluctuation (RMSF) values were calculated for the protein content in the complex (S2 Fig. and S3 Fig). Contact maps were obtained for residue pairs between the peptide and protein complex, and H-bond quantification was performed throughout the 300 ns of simulation (S4 Fig). Before binding free energy calculations, clustering was done on the production trajectories to obtain the most representative conformation using *cpptraj*’s hieragglo algorithm with an epsilon value of 2.0. The MM/PBSA method, using the MM-PBSA.py plugin from AmberTools24, was employed to calculate the binding free energy for each complex, utilizing frames from the longest trajectory section of the most representative cluster.

### Statistical analysis

A one-way ANOVA was performed using GraphPad Prism software (version 5.03) (GraphPad Software, San Diego, CA). ANOVA with Tukey’s post hoc statistical tests was used for pairwise comparisons among multiple groups. The significance levels are denoted as ***p < 0.001, **p < 0.01, and *p < 0.05.

## Supporting information

S1 Fig M13KE vector from 20 randomly selected phage clones. (DOCX)

S2 Fig Root Mean Square Deviation (RMSD) of the reach complex. (DOCX)

S3 Fig Root Mean Square Fluctuation (RMSF) of the protein for all complexes. (DOCX)

S4 Fig Heat map of atomic contacts between ZIKV-pE and linear peptides. (DOCX)

S5 Fig Contact lifetime of ZIKV-pE residues with linear peptide(s) across the trajectory. (DOCX)

S1 File ELISA assays data for selection of anti-ZIKV-pE peptides. (DOCX)
