# Supplementary material for: Discovery and computational characterization of ZIKV envelope-targeted peptides from a subtractive phage display library
Source: PLoS One. 2026 Jan 29;21(1):e0341602. doi: 10.1371/journal.pone.0341602 (PMC12854451; doi:10.1371/journal.pone.0341602)
Supplement: S1 Fig — (DOCX) [file pone.0341602.s001.docx]

**S1 Fig. M13KE vector from 20 randomly selected phage clones.** Obtained from the second and third biopanning rounds, were examined for integrity using electrophoresis to confirm their suitability for sequencing.

**
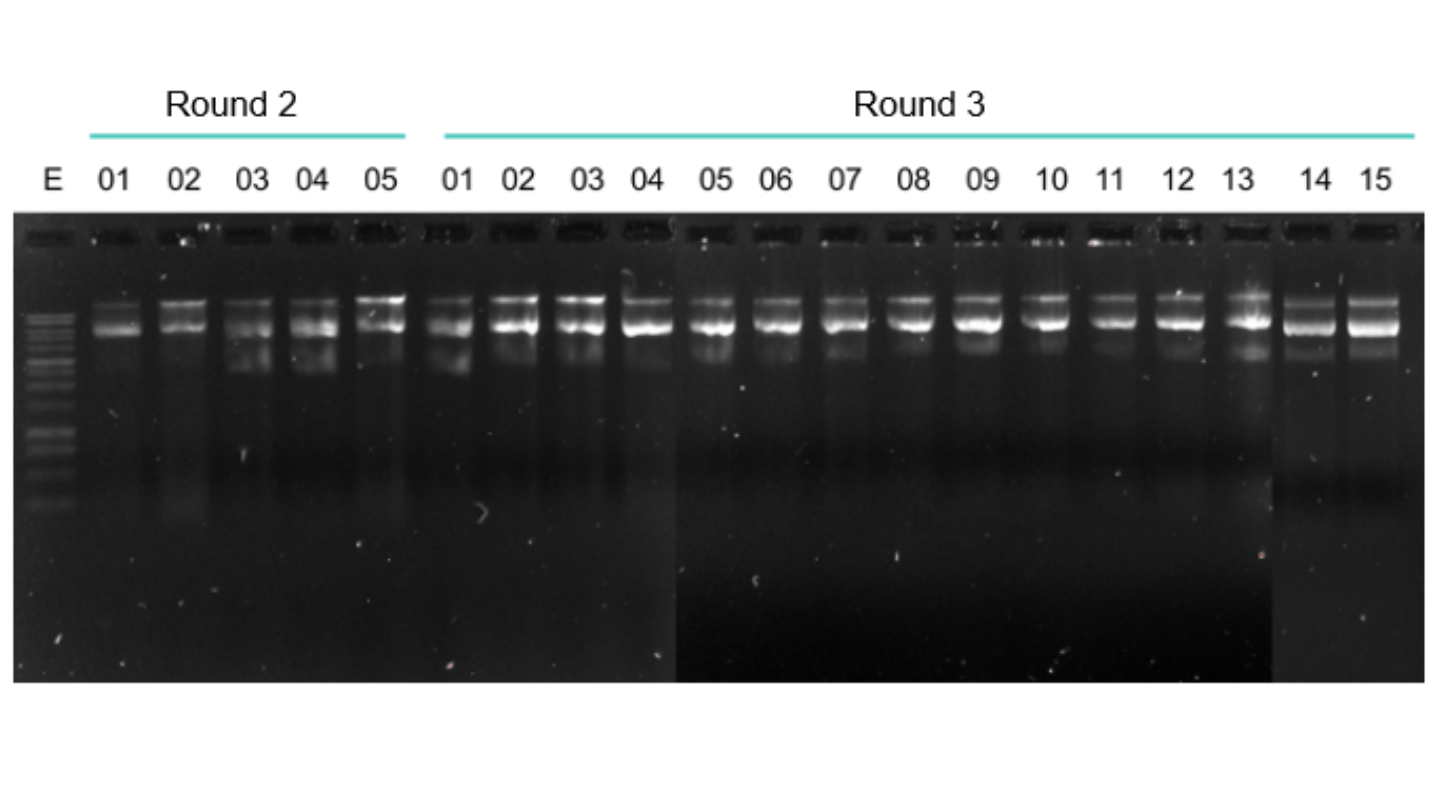
**
