# Supplementary material for: Discovery and computational characterization of ZIKV envelope-targeted peptides from a subtractive phage display library
Source: PLoS One. 2026 Jan 29;21(1):e0341602. doi: 10.1371/journal.pone.0341602 (PMC12854451; doi:10.1371/journal.pone.0341602)
Supplement: S2 Fig — (DOCX) [file pone.0341602.s002.docx]

**S2 Fig. Root Mean Square Deviation (RMSD) of the reach complex.** This figure presents the root mean square deviation (RMSD) values of the protein backbone for all complexes throughout the 300-ns molecular dynamics simulation. RMSD is a measure of the structural deviation from the initial conformation of the protein in the presence of different peptides over time. The plot allows for a comparison of the structural dynamics across the different complexes.**
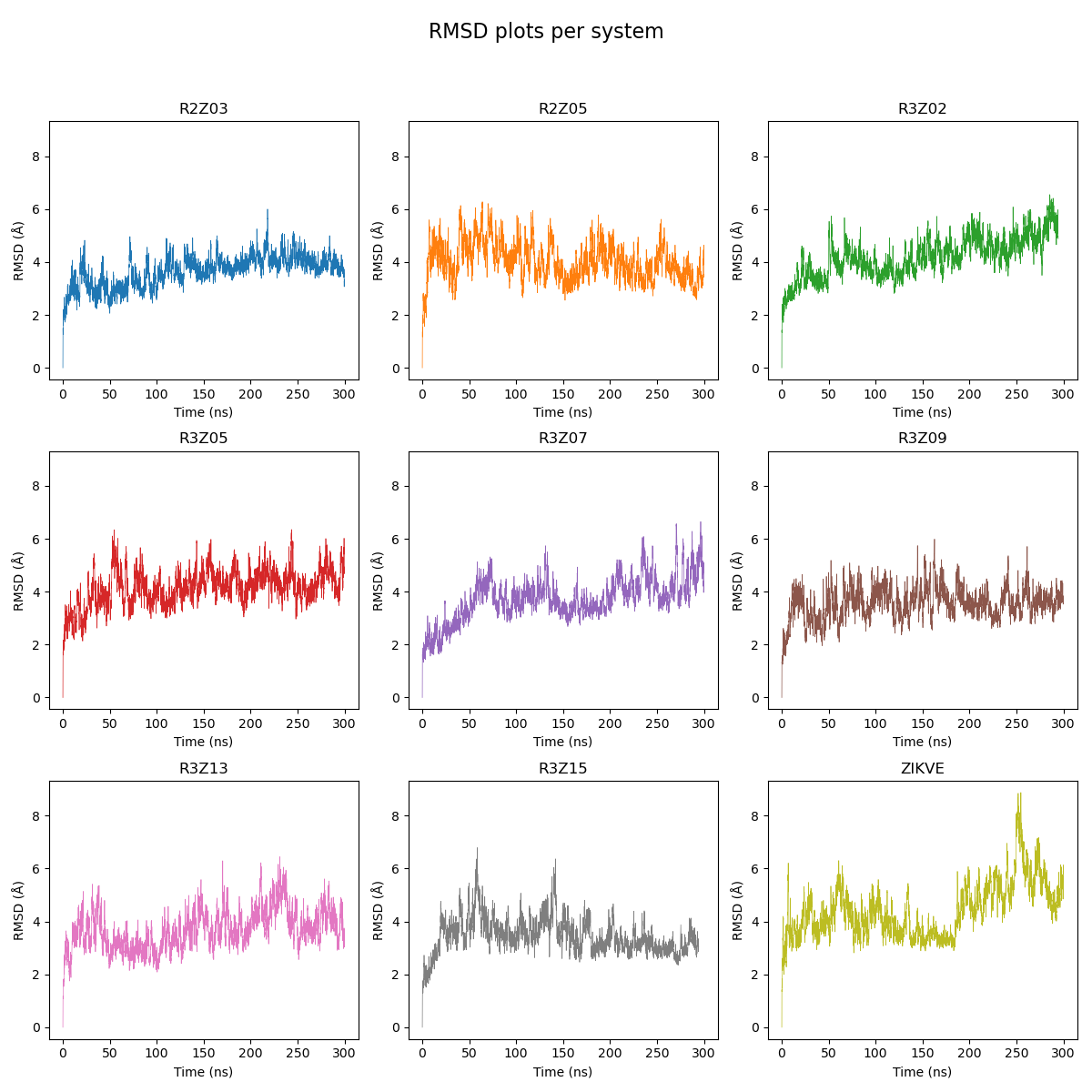
**
