# Supplementary material for: Discovery and computational characterization of ZIKV envelope-targeted peptides from a subtractive phage display library
Source: PLoS One. 2026 Jan 29;21(1):e0341602. doi: 10.1371/journal.pone.0341602 (PMC12854451; doi:10.1371/journal.pone.0341602)

**S3 Fig. Root Mean Square Fluctuation (RMSF) of the protein for all complexes.** This figure presents the RMSF values of the alpha carbons of the ZIKV-pE protein for all complexes, averaged over 300 ns of molecular dynamics simulation. RMSF measures the fluctuation of each residue from the initial conformation and averages. It is a method to visualize the flexible regions of a system. In this plot, we can see that the C- and N-terminal regions of both protein chains are the most flexible, as expected, and that all systems share a similar flexibility profile.


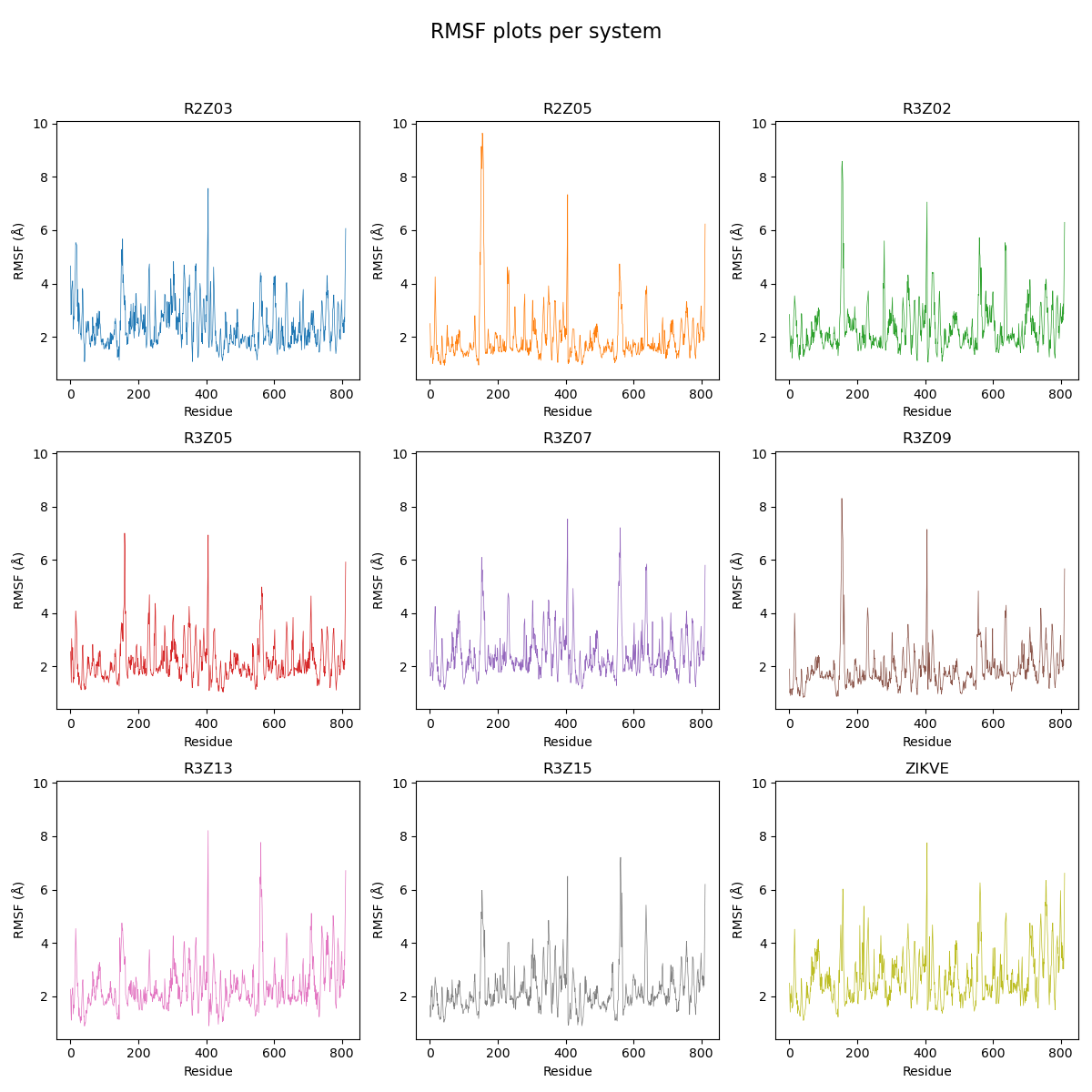

Supplement: S3 Fig — (DOCX) [file pone.0341602.s003.docx]
