# Supplementary material for: Discovery and computational characterization of ZIKV envelope-targeted peptides from a subtractive phage display library
Source: PLoS One. 2026 Jan 29;21(1):e0341602. doi: 10.1371/journal.pone.0341602 (PMC12854451; doi:10.1371/journal.pone.0341602)
Supplement: S4 Fig — (DOCX) [file pone.0341602.s004.docx]

**S4 Fig. Heat map of atomic contacts between ZIKV-pE and linear peptides.** This heat map visualizes the atomic contacts within 5 Å between the Zika virus envelope protein (ZIKV-pE) and the selected linear peptides. The x-axis represents residue intervals in the dimeric ZIKV-pE, while the color intensity indicates the frequency of atomic contacts within 5 Å between ZIKV-pE and selected peptides. This analysis helps identify key residues involved in the interaction between the viral protein and the peptides, providing insights into the binding interface and potential mechanisms of inhibition.

**
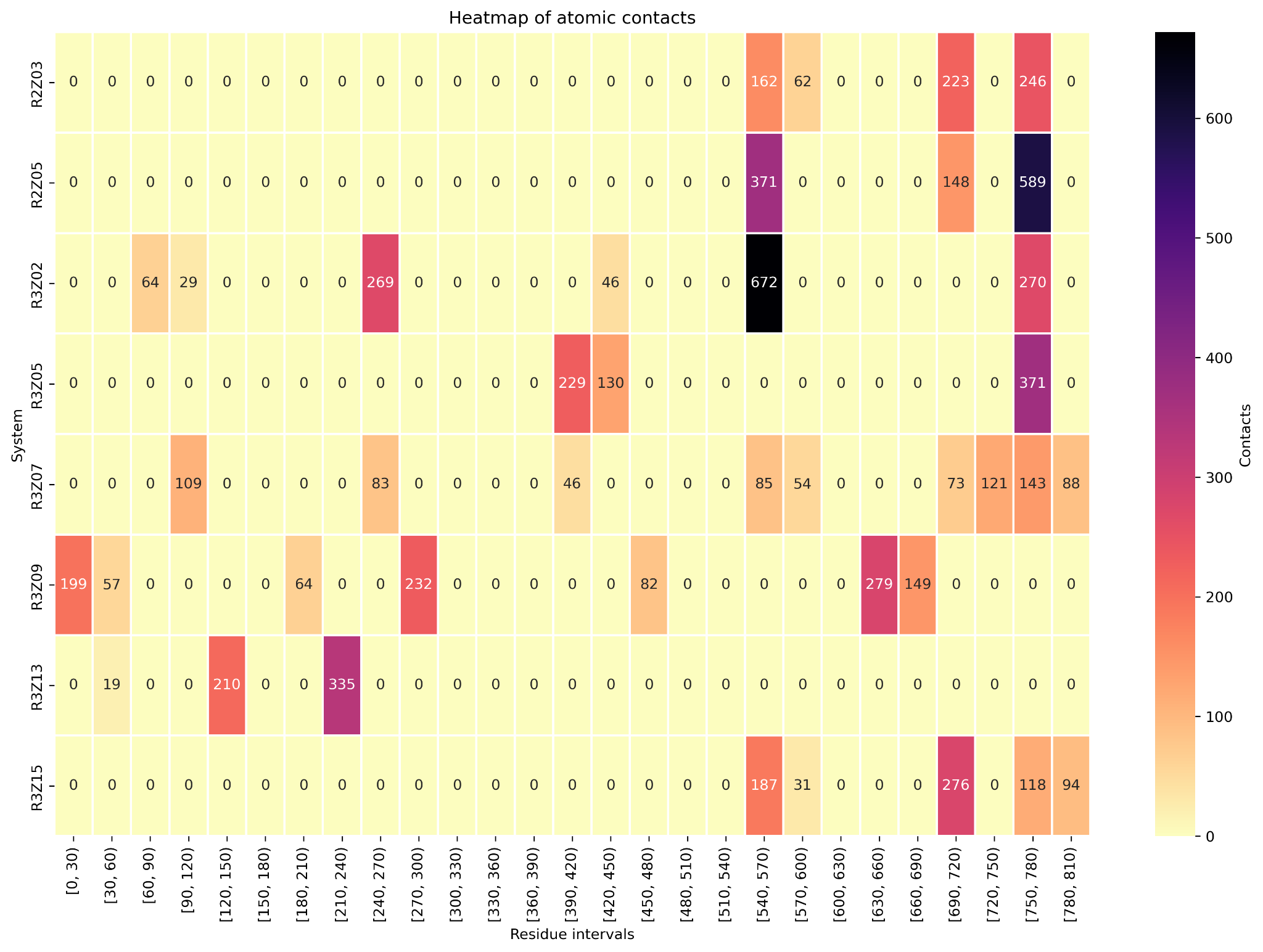
**
