# Supplementary material for: Discovery and computational characterization of ZIKV envelope-targeted peptides from a subtractive phage display library
Source: PLoS One. 2026 Jan 29;21(1):e0341602. doi: 10.1371/journal.pone.0341602 (PMC12854451; doi:10.1371/journal.pone.0341602)
Supplement: S5 Fig — (DOCX) [file pone.0341602.s005.docx]

**S5 Fig. Contact lifetime of ZIKV-pE residues with linear peptide(s) across the trajectory.** Residue contacts are considered within 5 Å of both molecules. The X-axis: Residues of ZIKV-pE (soluble ectodomain, residues 1–409, PDB ID: 5JHM). The domains are color-coded as follows: Domain I (DI, red), Domain II (DII, yellow), Domain III (DIII, blue), and the fusion loop (FL, purple), and the Y-axis: Fraction of the 300 ns MD trajectory during which a residue is in contact with the peptide. A value of 1.0 indicates continuous contact throughout the simulation, while lower values indicate transient or intermittent interactions.

**
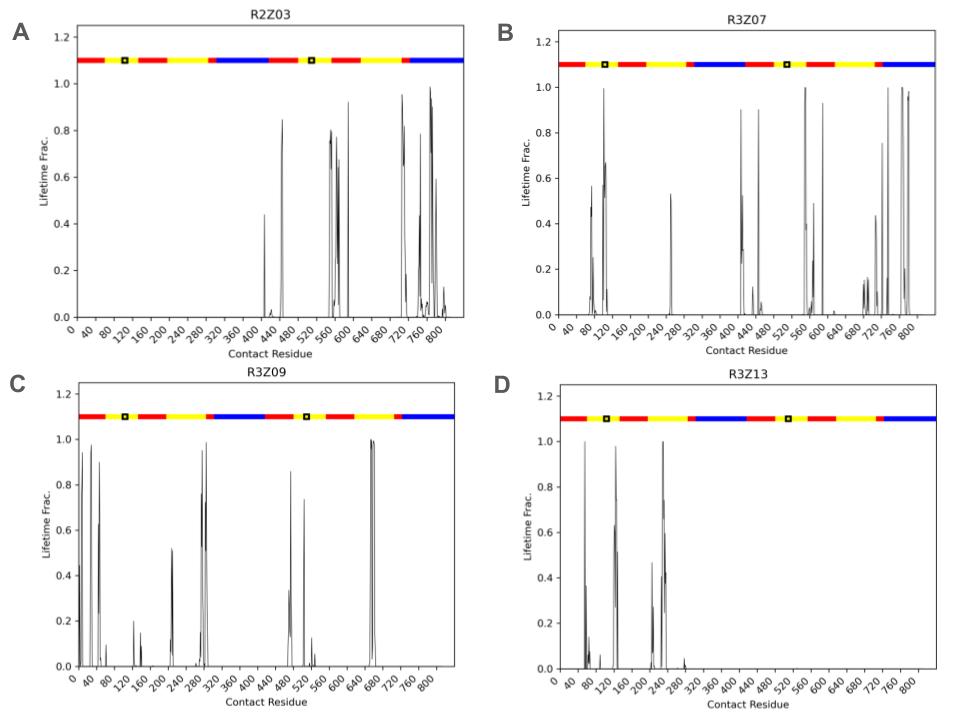
**
