# Supplementary material for: Discovery and computational characterization of ZIKV envelope-targeted peptides from a subtractive phage display library
Source: PLoS One. 2026 Jan 29;21(1):e0341602. doi: 10.1371/journal.pone.0341602 (PMC12854451; doi:10.1371/journal.pone.0341602)
Supplement: S1 File — (DOCX) [file pone.0341602.s006.docx]

***Phage-display for selection of anti-ZIKV-pE peptides.*** *Specificity of selected peptide-displaying phages for related ZIKV-pE or BSA were immobilized in wells for ELISA. Bound phages were detected using an antibody against M13 phage. The absorbance was detected at absorbance 450 nm.*

| **average Abs 450 nm** | | | **ZIKV pE** | | | **BSA 3 %** | | |
| --- | --- | --- | --- | --- | --- | --- | --- | --- |
| **Round** | **ZIKV pE** | **BSA 3 %** | **R1** | **R2** | **R3** | **R1** | **R2** | **R3** |
| **R0** | **0,175** | **0,072** | 0,187 | 0,160 | 0,177 | 0,057 | 0,070 | 0,090 |
| **R1** | **0,142** | **0,031** | 0,153 | 0,123 | 0,150 | 0,050 | 0,013 | 0,030 |
| **R2** | **0,789** | **0,309** | 0,783 | 0,780 | 0,803 | 0,374 | 0,247 | 0,307 |
| **R3** | **0,468** | **0,172** | 0,463 | 0,457 | 0,483 | 0,203 | 0,153 | 0,160 |

***Screening via ELISA assay of phage clones displaying peptides with binding capacity to ZIK-pE.*** *Recombinant ZIKV-pE was used as an antigen. All selected phages displaying peptides were challenged with ZIKV-pE, and the ELISA assay was performed with an Anti-M13-HRP antibody as the secondary antibody. The positive control (Ctrl +) consisted of a commercial monoclonal antibody against ZIKV-pE. An anti-M13 antibody was also used as a control to determine background recognition. The absorbance was detected at absorbance 450 nm.*

| **Individual clones** | **R1** | **R2** | **R3** |
| --- | --- | --- | --- |
| R2Z03 + ZIKV pE | 0.593 | 0.667 | 0.630 |
| R2Z03 + BSA | 0.390 | 0.363 | 0.410 |
| R2Z05 + ZIKV pE | 0.770 | 0.967 | 0.800 |
| R2Z05 + BSA | 0.390 | 0.380 | 0.347 |
| R3Z02 + ZIKV pE | 0.873 | 1.070 | 0.747 |
| R3Z02 + BSA | 0.297 | 0.313 | 0.270 |
| R3Z05 + ZIKV pE | 0.687 | 0.723 | 0.540 |
| R3Z05 + BSA | 0.327 | 0.227 | 0.270 |
| R3Z07 + ZIKVpE | 0.717 | 0.693 | 0.533 |
| R3Z07 + ZIKV BSA | 0.213 | 0.233 | 0.227 |
| R3Z09 + pE | 0.780 | 0.740 | 0.633 |
| R3Z09 + ZIKV BSA | 0.243 | 0.270 | 0.297 |
| R3Z13 + ZIKV pE | 0.827 | 1.033 | 0.813 |
| R3Z13 + BSA | 0.380 | 0.313 | 0.320 |
| R3Z15 + ZIKV pE | 0.823 | 0.880 | 0.803 |
| R3Z15 + BSA | 0.417 | 0.323 | 0.277 |
| Ctrl + (Ab anti ZIKV_pE) + ZIKV pE | 1.853 | 1.770 | 1.777 |
| Ctrl + (Ab anti ZIKV_pE) + BSA | 0.267 | 0.373 | 0.283 |
| Anti M13 + ZIKV pE | 0.420 | 0.510 | 0.430 |
| Anti M13 + BSA | 0.013 | 0.023 | 0.020 |
